# Altered Effective Connectivity of the Attentional Network in Temporal Lobe Epilepsy with EEG Data

**DOI:** 10.3390/bioengineering12040387

**Published:** 2025-04-04

**Authors:** Xiaojie Wei, Haojun Yang, Ruochen Dang, Bingliang Hu, Li Feng, Yuanyuan Xie, Quan Wang

**Affiliations:** 1 Key Laboratory of Spectral Imaging Technology, Xi’an Institute of Optics and Precision Mechanics (XIOPM), Chinese Academy of Sciences, Xi’ an 710119, China; weixiaojie19@mails.ucas.ac.cn (X.W.); dangruochen@opt.ac.cn (R.D.); hbl@opt.ac.cn (B.H.); 2 University of Chinese Academy of Sciences, Beijing 101408, China; 3 Key Laboratory of Biomedical Spectroscopy of Xi’ an, Xi’an Institute of Optics and Precision Mechanics (XIOPM), Chinese Academy of Sciences, Xi’ an 710119, China; 4 Department of Anesthesiology, Xiangya Hospital of Central South University, Changsha 410008, China; 15074937607@163.com; 5 Department of Neurology, Xiangya Hospital of Central South University, Changsha 410008, China; fenglihx@163.com

**Keywords:** electroencephalography, graphic theoretical analysis, attention network test, directed transfer function, temporal lobe epilepsy

## Abstract

Existing studies have shown that the attentional function of epilepsy is prone to be impaired. However, the characterization of brain connectivity behind this impairment remains uncertain. This study investigates attention-related brain connectivity in 92 patients with temporal lobe epilepsy and 78 healthy controls using a 32-channel EEG monitor during an attention network test. Compared to controls, patients showed reduced temporal–occipital connectivity in the alerting and orienting networks, but increased frontal–occipital connectivity in the executive network. Additionally, this study showed that patients and healthy individuals exhibited similar network topologies in the alerting and orienting networks, but the executive networks in patients showed altered topology properties, with a larger clustering coefficient in the theta band and a longer characteristic path length in the delta and theta bands. These findings reveal distinct characteristics of attention network connectivity in patients with temporal lobe epilepsy, offering valuable insights into the underlying mechanisms of epilepsy and providing clinical guidance for long-term monitoring and intervention.

## 1. Introduction

Epilepsy is a common chronic neurological disease of the brain characterized by recurrent, unprovoked seizures, impacting quality of life and increasing societal costs worldwide [1]. Temporal lobe epilepsy (TLE), a prevalent form of clinical epilepsy, is characterized by abnormal discharges from temporal lobe structures such as the hippocampus and amygdala. Neuropsychological studies have found that recurrent seizures of patients with TLE have varying degrees of problems related to cognition [2], such as memory [3], language [4], and attention deficits [5].

Attention is a complex process in which the brain processes, synthesizes, and distributes audio, visual, sensory, and other stimuli, which form the basis of all advanced cognition [1]. Based on the neurobiochemical mechanism of attention processing, Petersen and Posner proposed the theory of the attention network comprising three components: alerting, orienting, and executive control [6]. Fan et al. designed the attention networks test (ANT), which is widely used in the assessment of attention function in patients with epilepsy, schizophrenia, and healthy people [7,8,9]. Previous studies have demonstrated that patients with TLE have impairments in alerting [10,11], orienting [12], and executive networks [13]. However, such changes in functional connectivity, as well as the global topological properties of the brain networks in TLE, require further investigation.

Brain networks have been depicted in terms of functional connectivity by functional magnetic resonance imaging (fMRI) or electroencephalography (EEG). While prior attentional network research in TLE patients has mainly employed static resting-state fMRI (lacking directional information and dynamic sensitivity) [11] or EEG (typically without network topology analysis) [14], our study advances the field by examining multiple interacting attentional subnetworks, including the alerting, orienting, and executive networks.

The objective of the present study was to study the brain network mechanism behind the impaired attentional function in TLE patients. Understanding the neural mechanisms of epilepsy is crucial for achieving favorable treatment outcomes. To address this issue, the directed transfer function in functional connectivity analysis was used to assess the directional connectivity between different brain regions, and graph theoretic analysis was used to investigate the topological changes of the brain network, which helped to identify the characteristics of attentional brain networks in patients with TLE and provided new perspectives for the development of biomarkers based on the brain network.

## 2. Materials and Methods

### 2.1. Subjects

A total of ninety-two patients with TLE were recruited from Xiangya Hospital, Central South University, in Changsha, China, during the period from May 2022 to February 2023. For comparative analysis, seventy-eight healthy controls (HCs) were enrolled through local community recruitment and media promotion. The inclusion criteria for TLE patients were as follows: (1) diagnosis by psychiatrists through an assessment of clinical symptoms, MRI, and EEG results; (2) localization of the epileptogenic focus in the temporal lobe; and (3) age between 15 and 60. The exclusion criteria for all subjects included the following: (1) substance abuse; (2) risk of suicide; (3) pregnancy; (4) head injury; and (5) history of psychological disorders. This study was conducted in accordance with the Declaration of Helsinki and approved by the Ethics Committee of the Xiangya Hospital, Central South University (Approval No.202004081). Written informed consent was obtained from all participants prior to their inclusion in the study.

### 2.2. Behavioral Task Paradigms

**Figure 1:**
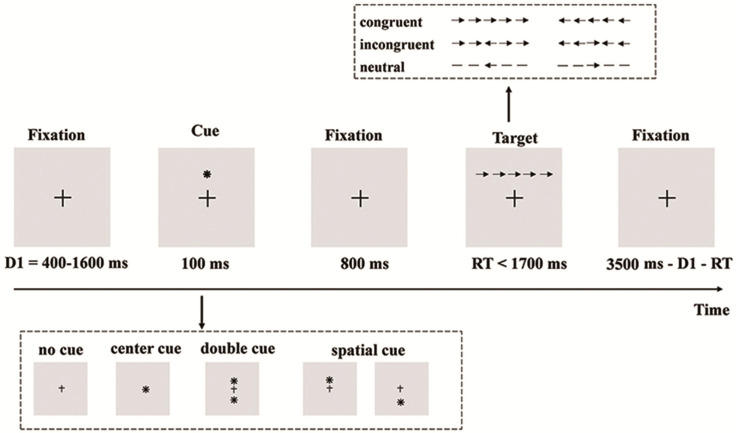
The experimental task. Participants performed an ANT task during EEG data recording. In each trial, a 100 ms cue was presented or the cross fixation remained unchanged (in the no-cue condition). After an 800 ms display of the fixation, the target appeared. Participants were required to respond within 1700 ms; otherwise, the target would disappear. Following this, a fixation cross of varying duration would appear. Each trial had a total duration of 4400 ms. * indicates the location where the target is about to appear.

The attention network test (ANT) paradigms developed by Fan et al. [15] were utilized in this study, as illustrated in Figure 1. The experiment was conducted in a room with low sound levels and dim lighting. Participants were seated comfortably approximately 60 cm away from a computer screen and directed to maintain focus on a stationary central cross throughout the experiment.

As shown in Figure 1, the ANT procedure involved four cue types (no cue, center cue, double cue, and spatial cue) and three target conditions (neutral, congruent, and incongruent). The center and double cues signal an imminent stimulus without location information, whereas the spatial cue indicates stimulus location. Each trial began with a fixation lasting between 400 and 1600 ms, followed by a 100 ms cue. After the cue, an 800 ms fixation appeared. Subsequently, the target was presented as a row of five arrows, with the central target arrow pointing either left or right, flanked by either congruent or incongruent arrows. Participants had to respond to the target direction within 1700 ms, after which the target and flankers disappeared.

The experiment included 12 trial combinations resulting from the cross of 4 cue types (no cue, center cue, double cue, spatial cue) and 3 target conditions (neutral, congruent, incongruent), distributed pseudo-randomly across three blocks, each containing 96 trials. Importantly, the preliminary experiments, including 24 practice trials, were conducted to ensure participants had grasped the task requirements. Participants were instructed to respond as quickly and accurately as possible throughout the test.

### 2.3. EEG Recording and Preprocessing

During the ANT task, EEG data were recorded using a BrainAmp MR system (BrainProducts GmbH, Gilching, Germany). The brain activity was obtained via 32 Ag/Cl electrode caps and displayed in real-time through a BP viewer. The EEG setup involved 32 electrodes positioned according to the 10–20 international system. The reference electrode was placed on the FCz site, while the ground electrode was placed on the AFz site. EEG signals were collected at a sampling rate of 500 Hz and maintained electrode impedance below 5 kΩ to ensure data quality.

EEG data preprocessing was performed using custom scripts written in MATLAB R2021b (The MathWorks, Inc., Natick, MA, USA), integrated with the EEGLAB toolbox [16]. The preprocessing pipeline encompassed several steps. First, we used custom scripts to import the raw EEG data into EEGLAB. This ensured the accurate configuration of electrode locations and montage settings. Next, to retain the desired signal frequencies, we applied a zero-phase finite impulse response (FIR) filter with a Hamming window (implemented via the pop_eegfiltnew function in the EEGLAB toolbox). Specifically, we employed a 0.1 Hz high-pass filter and a 70 Hz low-pass filter. Additionally, a 50 Hz notch filter was introduced to eliminate power line interference.

After filtering, we resampled the data to 250 Hz using EEGLAB’s pop_resample function. This step reduced the computational load. We then applied a common average reference (CAR), implemented by EEGLAB’s pop_reref function, to all channels. This minimized the spatial bias introduced by the original reference electrode.

The data were segmented into cue-locked epochs (400 ms before to 1700 ms after cue onset) and target-locked epochs (1300 ms before to 1700 ms after target onset) for decomposition. For each epoch, base correction was carried out using a pre-stimulus baseline period.

Finally, to identify and remove artifacts, we performed independent component analysis (ICA) with EEGLAB’s pop_runica function. Components related to ocular (blinks/saccades), electromyographic (EMG), and electrocardiographic (ECG) artifacts were identified through a combination of visual inspection of component topographies and time courses, as well as automated classification using the plug-in function ICLabel. Epochs with an amplitude exceeding ±100 μV in any channel were automatically discarded.

**Figure 2:**
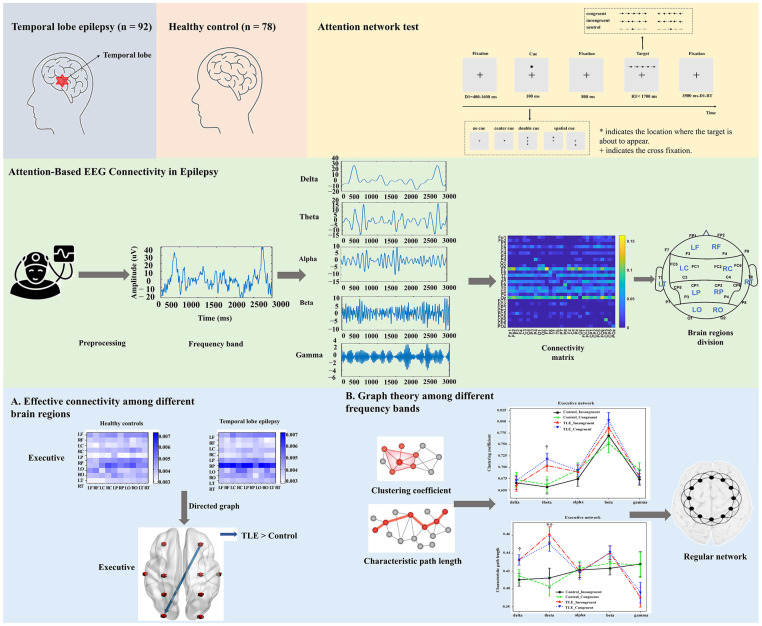
The schematic procedure for effective connectivity of the EEG signal and graphic theoretical metrics analysis.

Additionally, a medical professional screened for and removed epochs with abnormal epileptic activity, such as spikes or slow waves, to safeguard effective connectivity analysis from potential confounders. The overall analysis framework of this study after preprocessing is shown in Figure 2. The preprocessed EEG data were divided into five frequency bands. On this basis, we implemented effective connectivity from EEG signals between different brain regions using graphic theoretical metrics analysis.

### 2.4. Effective Connectivity Computation

Researchers have explored some methods for quantifying effectivity connectivity in multichannel EEGs, such as granger causality (GC) and partial directed coherence (PDC). The direct transfer function (DTF), a causality measure used in determination of brain connectivity patterns, was well affirmed and applied in many interpretations of experiments investigating brain connectivity [17]. The DTF algorithm, as described by Babiloni et al., hinges on the autoregressive coefficients of the multivariate autoregressive (MVAR) model to overcome the problem of non-stationarity of EEG data [18]. With two given EEG channels i and j considered, the information flow on the frequency f, calculated by the following equations, is a normalized representation of the transfer function H(f) [19].

(1) $$\gamma_{\mathrm{ij}}^{2}\left(f\right)=\frac{{\left|H_{\mathrm{ij}}\left(f\right)\right|}^{2}}{\sum_{m=1}^{k}{\left|H_{\mathrm{im}}\left(f\right)\right|}^{2}}$$

In Equation (1), $\gamma_{ij}^{2}(f)$ represents the DTF from channel j to channel i at frequency f. It measures how much of the signal at channel i is explained by the signal coming from channel i at that frequency f. $H_{ij}(f)$ denotes the transfer function describing how activity from channel j propagates to channel i at frequency f. It is typically derived from fitting a MVAR model to the time-series data and then converting the autoregressive coefficients into the frequency domain. Mathematically, H(f) is obtained by taking the inverse of the polynomial matrix that represents the MVAR model. $\sum_{m=1}^{k}|H_{im}(f){|}^{2}$ is the normalization term representing the total inflow to channel i from all channels (including j) in the model, ensuring that $\gamma_{ij}^{2}$ lies between 0 and 1. k denotes channel numbers. Higher values $\gamma_{ij}$ indicate stronger information flow or more robust directed connections.

Our study utilized the HERMES toolbox in MATLAB [20] to compute DTF values from preprocessed EEG data. Model order for each condition was empirically estimated using the Bayesian information criterion (BIC) across all participants, which balances model fit and complexity, avoiding overfitting while capturing essential neural dynamics. Based on derived model order, directional information flow was computed for all electrode pairs and participants, resulting in a DTF matrix with dimensions CH × CH × N × T. Here, CH represents the number of electrode nodes, N denotes the number of frequency bands, and T represents the number of time windows. Twenty-nine electrode nodes were used in the study, which covered key brain areas related to TLE, such as the frontal lobe, temporal lobe, parietal lobe, and occipital lobe, and these were electrode locations with high signal quality and less artifacts. Subsequently, we average the DTF values across the time windows in whole frequency bands, producing a connectivity matrix of 29 × 29 for each subject. Averaging across windows reduces transient noise while preserving sustained network interactions. Additionally, EEG data were decomposed into five canonical bands (delta: 0.5–4 Hz, theta: 4–8 Hz, alpha: 8–13 Hz, beta: 13–30 Hz, gamma: 30–50 Hz) to align with well-established neurophysiological correlates of cognition.

To mitigate the common source problem that can influence DTF calculations [21], we applied the current source density (CSD) method to spatially filter the EEG data using the CSD toolbox in MATLAB. The CSD method enhances spatio-temporal features of the EEG data while attenuating the long-distance effects caused by volume conduction [22,23,24].

### 2.5. Effective Connectivity Among the Brain Regions

Typically, the 29 electrodes were classified into ten brain regions to study casual interactions between different brain regions and pinpoint key areas in the attention network [25,26]. These regions were delineated as follows: left frontal (Fp1, F3), right frontal (Fp2, F4), left central (C3, FC1, FC5, and CP5), right central (C4, FC2, FC6, and CP6), left parietal (P3, CP1, and CP5), right parietal (P4, CP2, and CP6), left occipital (O1), right occipital (O2), left temporal (F7, T7, and P7), and right temporal (F8, T8, and P8). The effective connectivity from region k to region p was calculated using the formula

(2) $${\mathrm{DTF}}_{k\leftarrow p}=\frac{1}{\mathrm{MN}}\sum_{i\in K}\sum_{j\in P}r_{\mathrm{ij}}$$

In Equation (2), ${\mathrm{DTF}}_{k\leftarrow p}$ represents the average directed connectivity from region p to region k. Here, p and k represent two different sets of electrodes grouped by anatomical locale (e.g., frontal, temporal, etc.). $r_{ij}$ denotes the directed connectivity value from channel j (in region p) to channel i (in region k). In practice, $r_{ij}$ often corresponds to an average (or sum) of $\gamma_{ij}^{2}(f)$ values in formula (1) over a specific frequency band of interest. M is the number of channels in region k (where K denotes the channel set), and N signifies the number of channels in region p (with P representing the channel set). Dividing by MN yields the mean connectivity strength between the two regions.

The causal network efficiency of the three attention subnetworks is defined by the following equation [27,28]:

(3) $${{\mathrm{DTF}}_{\mathrm{Alerting}}=\mathrm{DTF}}_{\mathrm{no}\mathrm{cue}}-{\mathrm{DTF}}_{\mathrm{double}\mathrm{cue}},\;{\mathrm{DTF}}_{\mathrm{Orienting}}={\mathrm{DTF}}_{\mathrm{center}\;\mathrm{cue}}-{\mathrm{DTF}}_{\mathrm{spatial}\;\mathrm{cue}},\;{\mathrm{DTF}}_{\mathrm{Executive}}={\mathrm{DTF}}_{\mathrm{incongruent}}-{\mathrm{DTF}}_{\mathrm{congruent}},$$

The formulations ${\mathrm{DTF}}_{\mathrm{Alerting}}$, ${\mathrm{DTF}}_{\mathrm{Orienting}}$, ${\mathrm{DTF}}_{\mathrm{Executive}}$ separately represent the average DTF values across the respective attentional subnetworks. The average DTF values for different conditions are represented as follows: ${\mathrm{DTF}}_{\mathrm{no}\;\mathrm{cue}}$ for no-cue condition, ${\mathrm{DTF}}_{\mathrm{double}\;\mathrm{cue}}$ for double-cue condition, ${\mathrm{DTF}}_{\mathrm{center}\;\mathrm{cue}}$ for center-cue condition, ${\mathrm{DTF}}_{\mathrm{spatial}\;\mathrm{cue}}$ for spatial-cue condition, ${\mathrm{DTF}}_{\mathrm{incongruent}}$ for incongruent condition, and ${\mathrm{DTF}}_{\mathrm{congruent}}$ for congruent condition.

Since the seizures in TLE patients originate from the temporal lobe, particular attention was directed toward assessing the outflow values from the temporal lobe regions within the alerting and orienting networks. However, in the executive network, the outflow values from the frontal lobe regions are an important indicator, which are activated when tasks involve processing output control conflicts between different stimulus dimensions [29]. Finally, the brain-effective network was visualized using the BrainNet Viewer toolbox [30].

### 2.6. Graphic Theoretical Metrics Analysis

To study the precise brain networks of patients and controls, the brain was represented as a directed graph defined by a collection of nodes and directed links. Brain regions in the brain network are equivalent to nodes, while the values computed by DTF are equivalent to the directed links. The weight of such links can vary in the interval [0, 1], which represents the amount of information flowing between different brain regions. We set a threshold at the median of all connection strengths to convert the weighted graph to a binary graph, assigning a value of 1 to links above the median and 0 to those below. Subsequently, the binary-directed adjacency matrix was analyzed using graphic theoretical indicators [31]. Our study focused on two key graph theoretical metrics: clustering coefficient (CC) and characteristic path length (CPL). These metrics are extensively employed to characterize the organization and changes in brain networks in both healthy and epileptic brains [32] and are calculated as follow.

The CC is a measure of the degree to which nodes in the graph tend to cluster together. The average CC value of the network is the mean of the CC values for all nodes. CC reflects the degree of interconnection within specific network communities, measures interconnected clusters, and is related to the network’s local efficiency [33]. A higher CC indicates greater information transfer efficiency within these localized clusters.

The clustering coefficient C_i_ of node i is defined as

(4) $$C_{i}=\frac{E_{i}}{K_{i}(K_{i}-1)/2}$$

where E_i_ represents the number of connections that exist between the neighbors of node i, and K_i_ denotes the number of neighbor nodes of node i. Therefore, the denominator term $K_{i}(K_{i}-1)/2$ quantifies all possible connections between adjacent nodes. If node i has only one edge or no edges, then C_i_ is set to 0. The clustering coefficient C of the network is defined as the average clustering coefficient of all nodes in the network.

(5) $$C=\frac{1}{n}\sum_{i=1}^{n}Ci$$

In the formula, C refers to the clustering coefficient, which is used to measure the degree of cohesiveness in a network.

The CPL of a network, commonly called the average path length, represents the average of the shortest path lengths between all pairs of nodes in the network. This metric is a fundamental feature that describes a network’s topology and indicates how easily information can be transmitted through it [34,35]. A decreased CPL indicates a more efficient transmission of information throughout the network.

The characteristic path length L_i_ of node i is defined as

(6) $$L_{i}=\frac{1}{N-1}\sum_{i\neq j}\min\{L_{i,j}\}$$

where $\min\{L_{i,j}\}$ is the shortest absolute path length between node i and node j. The characteristic path length L_i_ of the node i is computed as the mean of the shortest absolute path lengths between pairs of nodes. The characteristic path length L of the network is defined as the average characteristic path length of all nodes in the network.

(7) $$L=\frac{1}{N}\sum_{i}L_{i}$$

In the formula, L represents the average characteristic path length, which measures the network’s average connectivity or overall routing efficiency.

The Brain Connectivity Toolbox (BCT) (http://www.nitrc.org/projects/bct/, accessed on 25 March 2024) was utilized to compute the graph’s theoretical metrics for assessing the binary-directed network’s topological characteristics [36].

### 2.7. Statistical Analysis

In this study, continuous variables are presented as means ± standard deviation (SD). To determine if there is a difference between the two groups (TLE patient, HC group), the independent sample t-test was conducted for continuous variables. The chi-square (χ^2^) test was used to compare count data among the groups. To control the probability of type I errors arising from multiple comparisons, the Bonferroni correction was applied to obtain the corrected *p*-values. To minimize the effects of trial variability, we averaged the data across multiple trials. Statistical analyses of demographic data and EEG effective connectivity were performed using SPSS software (version 22.0, SPSS Inc., Chicago, IL, USA). All statistical tests were conducted by bilateral test, and significance was set at corrected *p* < 0.05.

Mixed repeated measures ANOVAs were utilized to examine the graphic theoretical metrics (CC or CPL) of TLE patients within the attention network, considering two groups (TLE patient, HC group), two conditions (no cue vs. double cue, center cue vs. spatial cue, incongruent vs. congruent), and five frequency bands (delta, theta, alpha, beta, gamma). The group was treated as a between-subject factor, all trials for each subject were averaged, and each subject was treated as an independent sample. Frequency bands and conditions were treated as within-subject factors. If Mauchly’s test of sphericity was violated, the Greenhouse–Geisser correction was applied. In instances where no interaction effect between factors was observed in the ANOVA results, the main effects were evaluated directly. Alternatively, if an interaction effect was present, simple effects were analyzed. The partial eta squared (η^2^) was reported as a measure of effect size.

## 3. Results

### 3.1. Demographic Characteristics

This study included 92 patients with TLE and 78 HC. No significant differences between TLE patients and HC could be detected regarding sex, age, education level, household registration, or marital status. Detailed demographics are listed in Table 1.

### 3.2. Behavioral Characteristics

A comparative analysis was conducted on the behavioral characteristics from patients with TLE and the HC group during attention network tasks. The behavioral characteristics included the mean reaction time (MRT) and accuracy rates in responding to the tasks under various stimulus conditions, as shown in Table 2.

The results indicated a significant difference in MRT between the two groups across all experimental conditions (*p* < 0.05). Specifically, TLE patients exhibited longer MRT compared to the HC group in the single-task condition. Notably, while patients demonstrated shorter MRT in alerting and orienting network tasks, they displayed significantly longer MRT during executive network tasks. However, no significant between-group differences were found in accuracy rates (*p* > 0.05). This finding suggests that, although patients with TLE could understand and follow the task instructions accurately, their processing efficiency was compromised, particularly in higher-order cognitive demands associated with executive control.

### 3.3. Effective Connectivity

**Figure 3:**
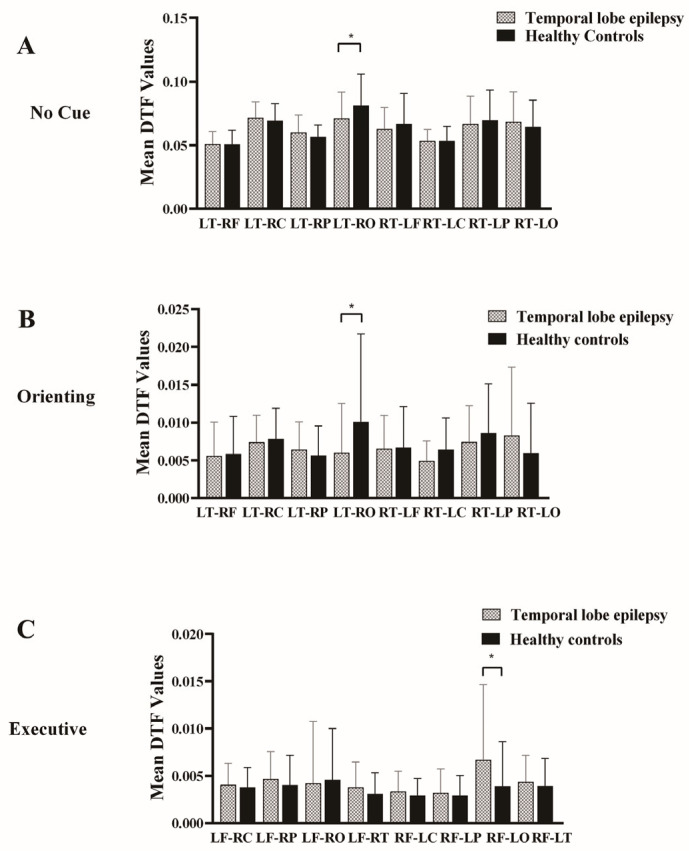
The comparison of the information outflow values between the TLE and HC group was shown under three attentional subnetwork conditions. (**A**) In the no-cue condition, patients with TLE exhibited decreased effective connectivity from the left temporal region to the right occipital region compared to the HC group. (**B**) In the orienting network condition, TLE patients also showed decreased effective connectivity from the left temporal region to the right occipital region compared to the HC group. (**C**) In the executive network condition, TLE patients demonstrated significantly increased effective connectivity from the right frontal region to the left occipital region. * represents *p* < 0.05.

To evaluate the differences of effective connectivity between the two groups, a two-sample *t*-test was conducted to investigate the information flow under each condition. In the no-cue condition, results showed that a notable reduction in effective connectivity in TLE patients was observed via post-Bonferroni correction than in the HC group (TLE patients, 0.071 ± 0.021; HC, 0.081 ± 0.025; *p* = 0.032; Figure 3A), with only one edge displaying significance. This edge connected from the left temporal region to the right occipital region. However, this difference was not found under the double-cue and alerting network conditions. The average connectivity matrices of different brain regions are shown in Figure S1.

We implemented the same analysis on the orienting network condition to evaluate the information flow; a notable reduction in effective connectivity in TLE patients was observed in a specific edge connecting the left temporal region to the right occipital region via post-Bonferroni correction, as compared to the HC group (TLE patients, 0.006 ± 0.006; HC, 0.010 ± 0.011; *p* = 0.032; Figure 3B).

**Figure 4:**
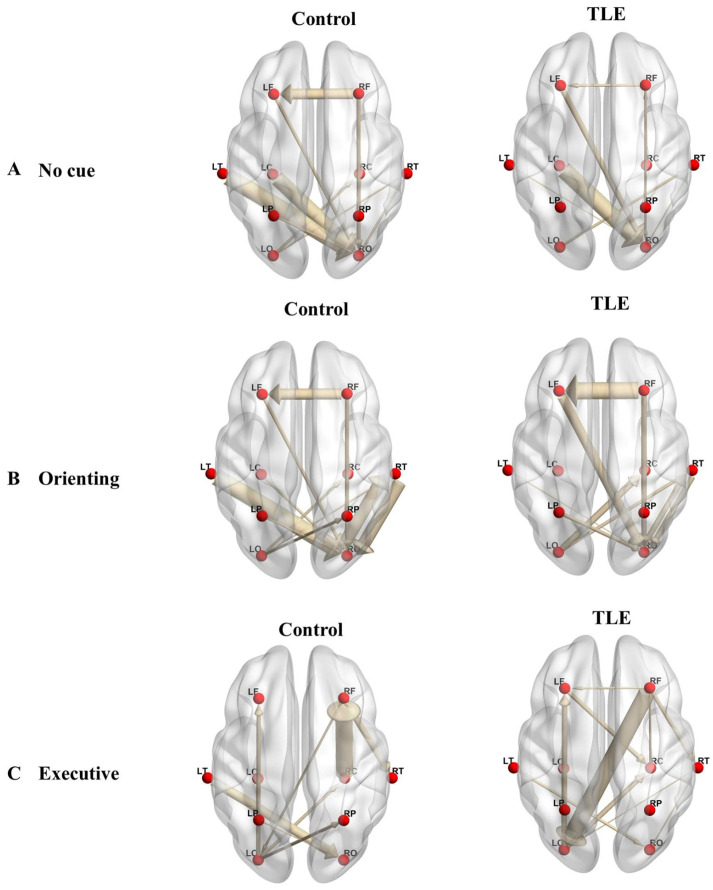
A directed graph incorporating all connections for both groups is presented under (**A**) no-cue condition, (**B**) orienting network, and (**C**) executive network. LF and RF represent the frontal regions of the left and right hemispheres, respectively; LC and RC denote the central regions of the left and right hemispheres; LP and RP indicate the parietal regions of the left and right hemispheres; LO and RO refer to the occipital regions of the left and right hemispheres; LT and RT represent the temporal regions of the left and right hemispheres.

In the executive network condition, we found that a marked enhancement in effective connectivity in TLE patients was noted in a singular edge connecting the right frontal region to the left occipital region following Bonferroni correction, in contrast to the HC group (TLE patients, 0.007 ± 0.008; HC, 0.004 ± 0.005; *p* = 0.048; Figure 3C). Figure 4 illustrates the connectivity graph incorporating all connections to visualize the distribution of different edges between the TLE patients and the HC group more intuitively.

### 3.4. Characteristics of Graphic Theoretical Metrics

To access the topological properties of the connectivity network, we derived the clustering coefficient and characteristic path length and implemented repeated measures ANOVA in three attentional subnetworks, as shown in Table S1.

**Figure 5:**
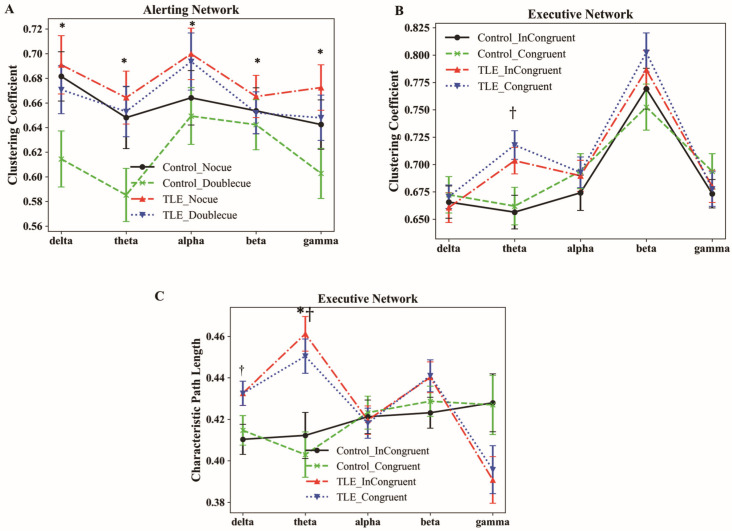
Difference of graphic theoretical metrics between two groups across different frequency bands. (**A**) The CC metric between the two groups under alerting network conditions across five frequency bands is presented. (**B**) The CC metric between the two groups under executive network conditions across five frequency bands is presented. (**C**) The CPL metric between the two groups under executive network conditions across five frequency bands is presented. Note: An asterisk (*) indicates a significant difference between the two conditions. A dagger (†) indicates a significant difference between the two groups.

Alerting network. The repeated measures ANOVA on CC with two groups (TLE, HC), two conditions (no cue, double cue), and five bands (delta, theta, alpha, beta, gamma) yielded a not significant interaction between condition and band (F_4,165_ = 1.726, *p* = 0.142, η^2^= 0.010). The group’s main effect was also insignificant (F_1,168_ = 2.018, *p* = 0.157, η^2^ = 0.012). Notably, the main effect of the condition was statistically significant (F_1,168_ = 5.332, *p* = 0.022, η^2^ = 0.031). Post hoc analysis indicated that the CC of the no-cue condition (represented by the red dot–dash line; Figure 5A) in the delta, theta, alpha, beta, and gamma bands was significantly larger than those of the double-cue condition in the TLE group (represented by the blue dotted line; Figure 5A). Similarly, in the HC group, the CC for the no-cue condition (represented by the black solid line; Figure 5A) was also significantly greater than that of the double-cue condition (represented by the green dashed line; Figure 5A) (ps < 0.05; Figure 5A).

The repeated measures ANOVA on CPL yielded a non-significant interaction between band and condition (F_4,165_ = 1.136, *p* = 0.333, η^2^ = 0.007). The main effect of the condition was also not significant (F_1,168_ = 2.965, *p* = 0.087, η^2^ = 0.017); similarly, the group’s main effect was also not significant (F_1,168_ = 1.343, *p* = 0.248, η^2^ = 0.008). Only the band’s main effect (F_4,165_ = 6.082, *p* = 0.002, η^2^ = 0.035) was statistically significant. Post hoc analyses indicated that the CPL of the theta and alpha bands was significantly larger than those of the gamma band (ps < 0.05).

Orienting network. The repeated measures ANOVA on CC with Mauchly’s test of sphericity, involving two groups (TLE, HC), two conditions (center cue, spatial cue), and five bands (delta, theta, alpha, beta, gamma), revealed a non-significant interaction between band and condition (F_4,165_ = 1.652, *p* = 0.159, η^2^ = 0.010). The main effect of the condition did not reach statistical significance (F_1,168_ = 0.492, *p* = 0.484, η^2^ = 0.003), and, similarly, the main effect of the group was also not significant (F_1,168_ = 2.096, *p* = 0.0150, η^2^ = 0.012). Notably, the main effect of the band was statistically significant (F _4,165_ = 4.063, *p* = 0.004, η^2^ = 0.024). Post hoc analyses indicated that the CC of the beta band was significantly larger than that of the delta, theta, and gamma bands (ps < 0.05).

The repeated measures ANOVA on CPL with Mauchly’s test of sphericity yielded a non-significant interaction between band and condition (F_4,165_ = 0.832, *p* = 0.472, η^2^ = 0.005). The main effect of the condition was also not significant (F_1,168_ = 0.022, *p* = 0.882, η^2^ < 0.001), and, similarly, the main effect of the group was also not significant (F_1,168_ = 1.510, *p* = 0.221, η^2^ = 0.009). Only the main effect of the band was statistically significant (F_4,165_ = 4.063, *p* = 0.004, η^2^ = 0.024). Post hoc analyses indicated that the CPL for the theta band was significantly larger than that for the gamma band (*p* < 0.05).

Executive network. The repeated measures ANOVA on CC (Figure 5B) with Mauchly’s test of sphericity, with two groups (TLE, HC), two conditions (congruent condition, incongruent condition), and five bands (delta, theta, alpha, beta, gamma), yielded a non-significant interaction between band and condition (F_4,165_ = 0.165, *p* = 0.956, η^2^ = 0.001). The main effect of the condition did not reach statistical significance (F_1,166_ = 2.293, *p* = 0.132, η^2^ = 0.014). Similarly, the group’s main effect was also insignificant (F_1,168_ = 2.737, *p* = 0.100, η^2^ = 0.016). However, the main effect of the band (F_4,163_ = 28.586, *p* < 0.001, η^2^ = 0.147) was statistically significant. Furthermore, there was a significant interaction between band and group (F_4,165_ = 2.687, *p* = 0.040, η^2^ = 0.016). Post hoc analyses indicated that the TLE patients’ CC in the theta band was significantly greater than that of the HC group (t = 3.540, *p* < 0.001). No significant differences between the two groups were observed in the remaining frequency bands. Specifically, in the theta band of the congruent condition, the CC of the TLE group (represented by the blue dotted line; Figure 5B) was significantly greater than that of the HC (represented by the green dashed line; Figure 5B). In the theta band of the incongruent condition, the CC of the TLE patients (represented by the red dot–dashed line; Figure 5B) was also significantly greater than that of the HC (represented by the black solid line; Figure 5B).

The repeated measures ANOVA on CPL (Figure 5C) with Mauchly’s test of sphericity was performed. The main effect of the condition (F_1,166_ = 0.122, *p* = 0.727, η^2^ = 0.001) was insignificant. And the main effect of the group (F_1,168_ = 2.737, *p* = 0.100, η^2^ = 0.016) was also insignificant. Only the main effect of the band (F_4,163_ = 28.586, *p* < 0.001, η^2^ = 0.147) was significant. Significant interactions were observed between band and condition (F_4,165_ = 8.260, *p* < 0.001, η^2^ = 0.047) and between band and group (F _4,165_ = 7.021, *p* = 0.003, η^2^ = 0.040).

Simple effects analyses were conducted between the condition and band, as shown in Table S2. These results indicated that in the incongruent condition, the CPL in the theta band was significantly greater than that in the congruent condition (t = 4.302, *p* < 0.001). Specifically, in the TLE patients, the CPL of the theta band in the incongruent condition (represented by the red dot–dashed line; Figure 5C) was longer than in the congruent condition (represented by the blue dotted line; Figure 5C). Similarly, in the HC, the CPL in the incongruent condition (represented by the black solid line; Figure 5C) was also longer than in the congruent condition (represented by the green dashed line; Figure 5C).

Simple effects analyses were also performed for the interaction between band and group, as presented in Table S2. The CPL in the delta band for TLE patients was longer than that of the HC group (t = 3.098, *p* = 0.027), and the CPL in the theta band for TLE patients was also longer than that of the HC group (t = 4.491, *p* < 0.001). Specifically, we found that in the delta and theta bands of the congruent condition, the CPL of the TLE patients (represented by the blue dotted line; Figure 5C) was longer than that of the HC (represented by the green dashed line; Figure 5C). In the incongruent condition, the TLE patients (represented by the red dot–dashed line; Figure 5C) also showed the longer CPL than the HC (represented by the black solid line; Figure 5C).

## 4. Discussion

This study deeply explored the attention networks’ effective connectivity and network topology in TLE patients. Compared with previous studies that primarily focused on a single network [11,14], our approach offered a more refined investigation of brain connectivity, uncovering distinct connectivity changes among various attentional networks (such as the alerting, orienting, and executive networks). In terms of disease-specific network reorganization, we found that TLE patients exhibited reduced temporal–occipital connectivity in the alerting and orienting networks, while displaying enhanced frontal–occipital connectivity in the executive network. Furthermore, this study integrated network topology with attentional networks in TLE, demonstrating that patients in the executive network exhibited a larger clustering coefficient in the theta band, as well as longer characteristic path lengths in the delta and theta bands. These findings not only enrich our understanding of attentional networks in TLE but also provide new insights into the underlying pathological mechanisms.

Analyses of effective connectivity in the alerting network of TLE patients revealed low connectivity between the temporal lobe and occipital lobe under the no-cue condition, suggesting the failure of a compensatory mechanism involving visual attention [37]. Under the no-cue and double-cue conditions, cognitive load differs significantly, with the former necessitating more cognitive resources [38]. In the no-cue condition, participants lack information about the upcoming stimulus, making the task more challenging and resulting in slower response times. Conversely, in the double-cue condition, the target presentation becomes more predictable, enhancing the expectancy regarding the stimulus presentation, which subsequently accelerates responses. These differences result in varying initial states of the brain when processing the forthcoming target stimuli, potentially influencing how the target stimuli are processed. Patients with TLE exhibited a decreased inflow of information from the temporal lobe to the occipital lobe when encountering unknown stimuli. This phenomenon may be linked to abnormal synchrony or reorganization of information pathways within the brain, leading to a smaller volume of information directed toward the occipital lobe. The temporal lobe has extensive fiber bundle connections with the frontal and occipital lobes and numerous reciprocal neural loops between the brain and the cerebellum. In TLE patients, repeated epileptic discharges in temporal lobe lesions can damage the frontal and occipital lobes, cerebellum, and brainstem through these connecting fibers. Consequently, patients may experience challenges effectively engaging these brain areas in response to stimuli, leading to decreased or impaired alertness [39].

Our analyses of effective connectivity in the orienting network of TLE patients revealed that TLE patients exhibited reduced flow values from the temporal lobe to the occipital lobe. Based on previous fMRI/MEG studies, the orienting network involves subcortical regions, including the lateral pulvinar of the thalamus and the superior colliculus [40]. TLE typically originates in the medial temporal lobe or other areas of the temporal lobe, and the epileptogenic zone may exhibit functional isolation, meaning that these regions have reduced communication with other brain areas. The presence of the epileptogenic zone may disrupt standard network connectivity, leading to restricted information transmission between brain regions. In particular, the reduced connectivity from the temporal lobe to the occipital lobe may reflect the impact of the epileptogenic zone on the visual cortex, impairing visual information processing. The temporal lobe is primarily associated with auditory processing, memory, and certain emotional functions, while the occipital lobe mainly involves visual processing. The reduced connectivity between the temporal and occipital lobes may indicate a cross-modal transmission impairment in patients with TLE.

We found differences in network organization between patients with TLE and the HC group during a visual task that requires executive control. It is speculated that abnormal connectivity strength in TLE patients may be related to excessive brain network activation during information processing [41], or maybe a compensatory effect arising from the network disturbances in patients with TLE, meaning the need for more attentional resources for a given task in patients with TLE [42]. Several studies support the above inference, with several resting-state fMRI studies revealing aberrant brain networks in patients with TLE [43,44]. Neuroimaging demonstrates the correlation between executive function and activity of the anterior cingulate cortex, prefrontal cortex, partial basal ganglia, and thalamus. Our findings expand on this by showing that executive function activation involves the frontal and occipital lobes, aligning with research that executive function depends on a coordinated network across multiple brain regions [45].

The observed dual pattern of connectivity changes (increased frontal–occipital connectivity in the executive network and reduced temporal–occipital connectivity in alerting/orienting networks) likely reflects a combination of compensatory mechanisms and pathology-driven network reorganization in TLE. Specifically, the increased frontal–occipital connectivity within the executive network likely represents a compensatory enhancement [46]. Here, the prefrontal cortex may upregulate its communication with occipital regions to mitigate deficits in high-level cognitive functions—such as decision-making and executive control—caused by the underlying epileptic pathology. In contrast, the reduced temporal–occipital connectivity in the alerting and orienting networks seems to be driven primarily by direct pathological disruptions, including hippocampal sclerosis and temporal lobe atrophy [47], which impair the processing of external stimuli and spatial orientation. Thus, while the executive network may strengthen its connections as an adaptive response to maintain cognitive performance, the alerting and orienting networks suffer connectivity suppression due to the direct impact of epileptic activity. Overall, these findings suggest that the brain employs both compensatory mechanisms and network reorganization to cope with the diverse effects of TLE on different functional systems.

Our findings indicate that TLE patients exhibit significantly reduced temporal–occipital connectivity in the alertness network, which parallels an fMRI study [11] that reported decreased activation in the right occipital lobe and cerebellum, suggesting that occipital dysfunction, a core component of visual processing, is central to alertness network deficits in TLE. In contrast, our study revealed enhanced frontal–occipital connectivity and altered network topology in the executive network, as evidenced by increased theta band clustering coefficients and prolonged characteristic path lengths. While an EEG study by Ren et al. [25] found significantly weakened theta band connectivity within the executive control network, correlating with behavioral performance, our topological metrics further elucidate the dynamic mechanisms underlying these connectivity changes. Moreover, although Ren et al. [14] also reported weakened overall executive network connectivity—especially between the frontal lobe and other regions—in epilepsy patients with depression, our study, focusing specifically on TLE patients without depression, underscores distinct pathological profiles that may account for these differences.

Our findings suggest that the preservation of network topology in the alerting and orienting networks suggests that basic attentional processes remain relatively intact in TLE patients, whereas higher-order executive functions are more affected. Moreover, the altered topology of the executive network, characterized by a higher clustering coefficient in the theta band and a longer characteristic path length in the delta and theta bands, reflects impaired information integration and transmission efficiency. These changes may underlie deficits in executive control, including difficulties in task switching and conflict resolution. These changes also confirm that, compared with HC patients, the network topology of TLE patients is more regular, which is consistent with the conclusions of previous studies using graph theory analysis [48].

Our study makes several key contributions. First, it reveals that TLE patients exhibit the following distinct alterations in attention network connectivity: a significant reduction in temporal–occipital connectivity in the alerting and orienting networks and enhanced frontal–occipital connectivity in the executive network. Second, while the topological organization of the alerting and orienting networks remains comparable to that of healthy controls, the executive network in TLE patients shows notable changes, including a larger clustering coefficient in the theta band and longer characteristic path lengths in the delta and theta bands. Third, combining multi-frequency functional connectivity with complex network topology analysis overcomes the limitations of traditional single metrics and comprehensively characterizes the oscillatory and integrative properties of the TLE network.

This study had several limitations that warrant consideration. Firstly, we observed some atypical connectivity patterns, particularly in the executive network, which warrant further investigation. Secondly, several unresolved issues remain, such as the potential influence of medication, seizure severity, and other clinical factors on network topology. Future studies should address these gaps by collecting a broader dataset and explicitly incorporating these potential influences into the analytical framework. Thirdly, the low spatial resolution of EEG renders the precise localization of nodes somewhat imprecise. For a more comprehensive understanding and validation of our findings, employing a multimodal approach—particularly integrating EEG with fMRI data—is advisable.

## 5. Conclusions

Our study investigated the brain network mechanisms behind patients’ impaired attentional functions through an in-depth study of EEG signals using functional connectivity techniques combined with graph theory analysis. The effective connectivity of the network was assessed using frequency-domain Granger causality analysis, and the local and global topology of the entire network was evaluated using clustering coefficients and characteristic path lengths. TLE patients showed reduced connectivity between temporal and occipital regions in the alerting and orienting networks. However, increased connectivity between the frontal and occipital lobes was found in the executive network in patients with TLE. In the alerting and orienting networks, TLE patients and the healthy individuals showed similar network topologies. However, in the executive network, TLE patients showed tight connectivity between neighboring nodes, while relying more on intermediate connections for communication at distant nodes in the theta band. This observation enhances our understanding of the interactions between brain regions in the attentional network and the topology of the impaired attention network in TLE patients.

## Figures and Tables

**Table 1:** Demographic characteristics of all subjects.

Group		TLE Patients (*n* = 92)	HC (*n* = 78)	Statistical Value *t*-Value/*χ*^2^-Value	*p*-Value
Sex ^a^	Female/Male	39/53	44/34	3.320	0.068
Age ± SD (years) ^b^		30.80 ± 12.82	27.41 ± 10.55	1.857	0.065
Education level ^a^	Elementary school	6	2	4.254	0.373
	Junior high school	19	12		
	High school or vocational school	26	22		
	Associate degree	20	15		
	College degree or higher	21	27		
Household registration ^a^	Urban/Rural	57/35	55/23	0.241	0.241
Marital status ^a^	Married/Unmarried	48/44	40/38	0.013	0.908
Duration of illness ± SD (years)		7.53 ± 9.05	—		—
Duration of episodes ± SD (minutes)		2.12 ± 1.71	—		—

Notes: Statistical analyses: ^a^ Chi-square test between TLE patients and HC group; ^b^ two-sample *t*-test between TLE patients and HC group.

**Table 2:** Differences in MRT and accuracy rate between the two groups.

	MRT (Millisecond)			Accuracy Rate		
	TLE Patients	HC Group	*p*-Value	TLE Patients	HC Group	*p*-Value
No cue	645.45 ± 112.59	573.36 ± 83.27	<0.001	0.970 ± 0.070	0.988 ± 0.015	0.539
Double cue	609.71 ± 122.31	529.36 ± 82.55	<0.001	0.971 ± 0.068	0.985 ± 0.020	0.914
Center cue	614.41 ± 119.66	538.71 ± 85.13	<0.001	0.970 ± 0.067	0.980 ± 0.019	0.796
Spatial cue	575.84 ± 134.61	483.75 ± 86.71	<0.001	0.977 ± 0.550	0.990 ± 0.016	0.181
Congruent	585.47 ± 124.91	504.92 ± 82.66	<0.001	0.987 ± 0.050	0.997 ± 0.008	0.085
Incongruent	687.36 ± 148.68	586.27 ± 90.94	<0.001	0.943 ± 0.130	0.968 ± 0.033	0.847
Alerting network	35.73 ± 30.35	44.00 ± 23.44	0.047	-	-	-
Orienting network	38.56 ± 34.08	54.95 ± 24.60	0.001	-	-	-
Executive network	101.88 ± 61.52	81.35 ± 32.35	0.018	-	-	-

## Data Availability

The data supporting the findings of this study are available upon request from the corresponding author. The data were not publicly available because of privacy or ethical restrictions.

## Supplementary Materials

The following supporting information can be downloaded at https://www.mdpi.com/article/10.3390/bioengineering12040387/s1, Figure S1: The connectivity matrices of mean DTF for all patients and healthy controls were presented under (A) alerting network, (B) orienting network, and (C) executive network conditions; Table S1: The ANOVA results for graphic theoretical metrics under the attentional subnetwork condition; Table S2: Simple effect analysis of the interaction under the executive network condition.

## Abbreviations

The following abbreviations are used in this manuscript:

TLE	Temporal lobe epilepsy
ANT	Attention networks test
fMRI	Functional magnetic resonance imaging
EEG	Electroencephalography
HC	Healthy control
FIR	Finite impulse response
CAR	common average reference
ICA	Independent component analysis
EMG	Electromyogram
ECG	Electrocardiogram
GC	Granger causality
PDC	Partial directed coherence
DTF	Direct transfer function
MVAR	Multivariate autoregressive
BIC	Bayesian information criterion
CSD	Current source density
CC	Clustering coefficient
CPL	Characteristic path length
BCT	Brain Connectivity Toolbox
SD	Standard deviation
MRT	mean reaction time
