# Supplementary material for: Altered Effective Connectivity of the Attentional Network in Temporal Lobe Epilepsy with EEG Data
Source: Bioengineering (Basel). 2025 Apr 4;12(4):387. doi: 10.3390/bioengineering12040387 (PMC12025012; doi:10.3390/bioengineering12040387)
Supplement: Supplementary file 1 [file bioengineering-12-00387-s001.zip › bioengineering-3521582-supplementary.pdf]

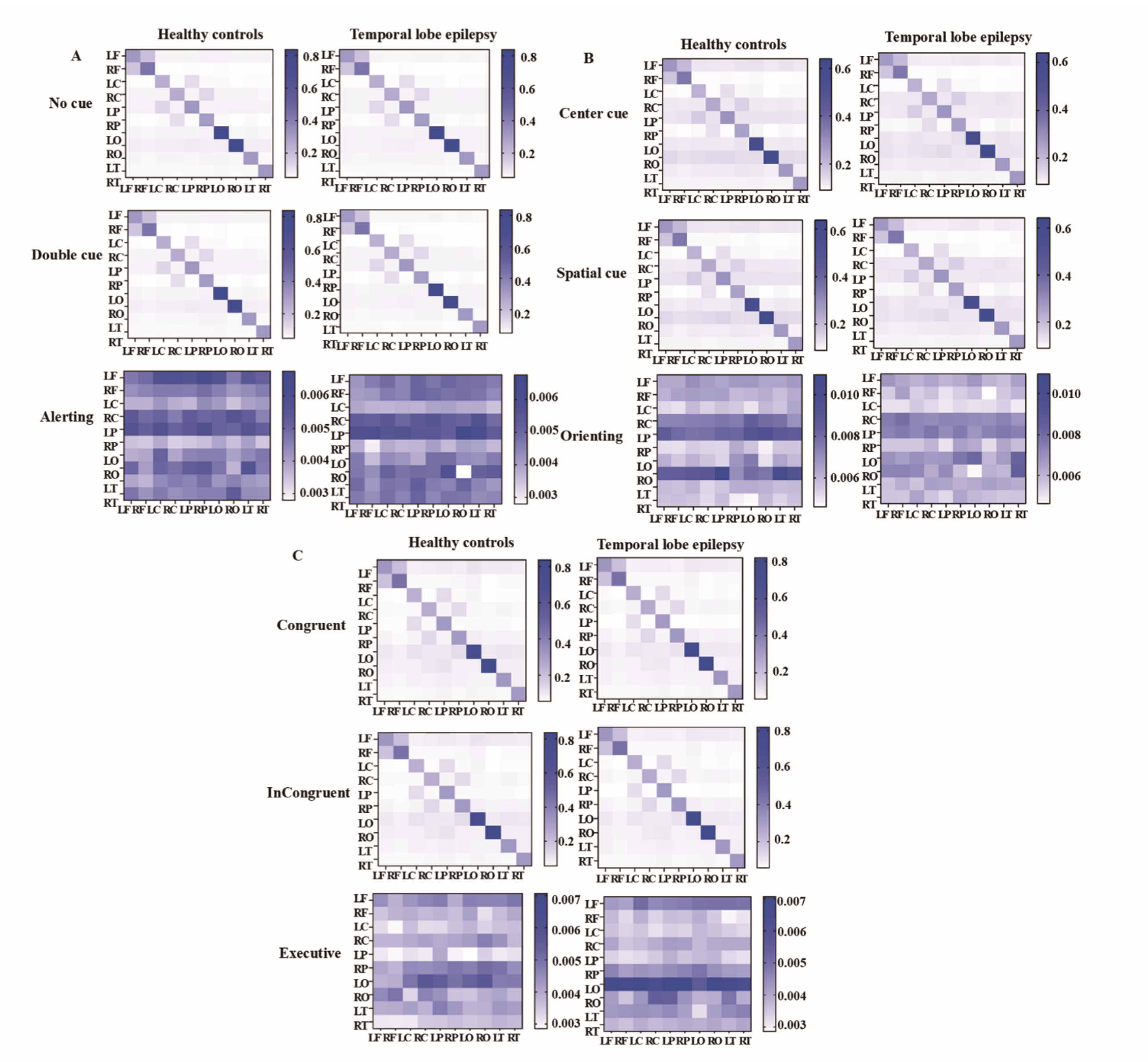

Figure S1. The connectivity matrices of mean DTF for all patients and healthy controls were presented under (A) alerting network, (B) orienting network, and (C) executive network condition.

Table S1. The ANOVA results for graphic theoretical metrics under the attentional subnetwork condition.

Network	Index	CC			CPL		
		F- value	*p*-value	Effect size *η2*	*F*- value	*p*- value	Effect size *η2*
Alerting network	Condition	5.332	**0.022**[a]	0.031	2.965	0.087	0.017
	Band	2.271	0.066	0.013	6.082	**0.002**[a]	0.035
	Group	2.018	0.157	0.012	1.343	0.248	0.008
	Condition * Band	1.726	0.142	0.010	1.136	0.333	0.007
	Condition * Group	3.299	0.071	0.019	0.462	0.498	0.003
	Band * Group	0.877	0.470	0.005	0.585	0.673	0.003
	Band * Condition* Group	0.828	0.503	0.005	0.344	0.788	0.002

Orienting network	Condition	0.492	0.484	0.003	0.022	0.882	< 0.001
	Band	4.063	**0.004**[a]	0.024	3.539	**0.026**[a]	0.021
	Group	2.096	0.150	0.012	1.510	0.221	0.009
	Condition * Band	1.652	0.159	0.010	0.832	0.472	0.005
	Condition * Group	1.639	0.202	0.010	0.519	0.472	0.003
	Band * Group	0.126	0.964	0.001	0.387	0.697	0.002
	Band * Condition* Group	1.321	0.261	0.008	0.435	0.718	0.003
Executive network	Condition	2.293	0.132	0.014	0.122	0.727	0.001
	Band	28.586	**< 0.001**[a]	0.147	5.527	**< 0.001**[a]	0.118
	Group	2.737	0.100	0.016	1.557	0.214	0.009
	Condition * Band	0.165	0.956	0.001	8.260	**< 0.001**[a]	0.047
	Condition * Group	2.687	**0.040**[a]	0.016	7.021	**0.003**[a]	0.040
	Band * Group	0.001	0.978	0.000	0.334	0.564	0.002
	Band * Condition* Group	0.965	0.4226	0.006	1.389	0.243	0.008

Note: [a] denotes p-value < 0.05.

Table S2. Simple effect analysis of the interaction under the executive network condition.

**Index**	**Interaction**	**Band**	**TLE patients**	**HC group**	***p*-value**
CC	band * group	Delta	0.67 ± 0.01	0.67 ± 0.01	0.864
		Theta	0.72 ± 0.01	0.66± 0.01	**< 0.001**[b]
		Alpha	0.69 ± 0.01	0.68 ± 0.01	0.613
		Beta	0.80 ± 0.02	0.76 ± 0.02	0.099
		Gamma	0.68 ± 0.01	0.68 ± 0.01	0.759
**Index**	**Interaction**	**Band**	**Incongruent**	**Congruent**	***p*-value**
CPL	condition * band	Delta	0.42 ± 0.01	0.42 ± 0.01	0.273
		Theta	0.44 ± 0.01	0.43 ± 0.01	**< 0.001**[b]
		Alpha	0.42 ± 0.01	0.42 ± 0.01	0.906
		Beta	0.43 ± 0.01	0.44 ± 0.01	0.148
		Gamma	0.41 ± 0.01	0.41 ± 0.01	0.485
**Index**	**Interaction**	**Band**	**TLE patients**	**HC group**	***p*-value**
CPL	band * group	Delta	0.43 ± 0.01	0.41 ± 0.01	0.027[b]
		Theta	0.46 ± 0.01	0.41 ± 0.01	**< 0.001**[b]
		Alpha	0.42 ± 0.01	0.42 ± 0.01	0.748
		Beta	0.44 ± 0.01	0.43 ± 0.01	0.162
		Gamma	0.39 ± 0.01	0.43 ± 0.01	0.055

Note: [b] denotes p-value < 0.05
